# Lymphovascular invasion is associated with poor long-term outcomes in patients with pT1N0-3 or pT2-3N0 remnant gastric cancer: a retrospective cohort study

**DOI:** 10.1186/s12957-024-03371-z

**Published:** 2024-04-05

**Authors:** Shutaro Sumiyoshi, Takuma Ohashi, Takeshi Kubota, Keiji Nishibeppu, Kaho Owada, Jun Kiuchi, Hiroki Shimizu, Tomohiro Arita, Daisuke Iitaka, Yusuke Yamamoto, Hirotaka Konishi, Ryo Morimura, Kenji Watanabe, Yoshiaki Kuriu, Atsushi Shiozaki, Hisashi Ikoma, Hitoshi Fujiwara, Nobuki Yamaoka, Eigo Otsuji

**Affiliations:** 1 Division of Digestive Surgery, Department of Surgery, Kyoto Prefectural University of Medicine, 465 Kajii-Cho, Kamigyo-Ku, Kyoto, 602-8566 Japan; 2 Department of Surgery, Kyoto Chubu Medical Center, 25 Yagiueno, Yagi-Cho, Nantan-Shi, Kyoto, 629-0197 Japan

**Keywords:** Remnant gastric cancer, Lymphovascular invasion, Gastrectomy, Prognosis, Surgery

## Abstract

**Background:**

Lymphovascular invasion (LVI) is a poor prognostic factor in various malignancies. However, its prognostic effect in remnant gastric cancer (RGC) remains unclear. We examined the correlation between LVI and disease prognosis in patients with T1N0-3 or T2-3N0 RGC in whom adjuvant chemotherapy was not indicated and a treatment strategy was not established.

**Methods:**

We retrospectively analyzed patients with T1N0-3 and T2-3N0 RGC who underwent curative surgery at the Kyoto Prefectural University of Medicine between 1997 and 2019 and at the Kyoto Chubu Medical Center between 2009 and 2019.

**Results:**

Fifteen of 38 patients (39.5%) with RGC were positive for LVI. Patients with LVI had a significantly poorer prognosis for both overall survival ([OS]: *P* = 0.006) and recurrence-free survival ([RFS]: *P* = 0.001) than those without LVI. Multivariate analyses using the Cox proportional hazards model revealed LVI as an independent prognostic factor affecting OS (*P* = 0.024; hazard ratio 8.27, 95% confidence interval:1.285–161.6) and RFS (*P* = 0.013; hazard ratio 8.98, 95% confidence interval:1.513–171.2).

**Conclusions:**

LVI is a prognostic factor for patients with T1N0-3 or T2-3N0 RGC. Evaluating LVI may be useful for determining treatment strategies for RGC.

**Supplementary Information:**

The online version contains supplementary material available at 10.1186/s12957-024-03371-z.

## Introduction

Remnant gastric cancer (RGC) refers to all cancers that arise from the remnant stomach after gastrectomy, regardless of the initial disease or type of gastrectomy [1]. The incidence of RGC after distal gastrectomy is reportedly 1–8% [2–4]. The clinical entity of RGC was first described by Balfour in 1922, defining it as the occurrence of carcinoma in the remnant after operation for benign disease [5]. Currently, there is no global consensus on the exact definition of RGC, especially regarding the interval after gastrectomy for the first malignant disease. Reports vary from those encompassing the immediate postoperative period to those that include only the interval more than 10 years later [6, 7]. Notably, gastrectomies for peptic ulcer disease have dramatically declined in recent decades, leading to a reduction in the number of patients undergoing gastrectomies for benign diseases. However, owing to the improved prognosis following gastric cancer treatment, individuals with a history of malignancy are increasingly susceptible to developing RGC [8]. Therefore, understanding the clinical features of RGC is critical for developing optimal surgical and treatment strategies. Yet, the information available regarding RGC treatment in the literature is limited.

Lymphovascular invasion (LVI) indicates the presence of tumor cells inside the blood or lymphatic vessels within the primary tumor. LVI is independently associated with poor long-term outcomes in various malignant tumors [9–11]. In patients with colorectal cancer, LVI is a high-risk factor for recurrence and serves as a criterion for postoperative adjuvant chemotherapy [12]. However, in gastric cancer, LVI is clinically useful only for evaluating the curability following endoscopic resection and not as a criterion for adjuvant chemotherapy [13]. Furthermore, the clinical significance of LVI in RGC remains unclear, and LVI may be a high-risk factor for recurrence in RGC, even in T1N0-3 or T2-3N0 patients who do not qualify for adjuvant chemotherapy under current guidelines due to favorable prognosis in gastric cancer. This study aimed to evaluate the prognostic impact of LVI on T1N0-3 or T2-3N0 RGC.

## Methods

### Patients

**Fig. 1:**
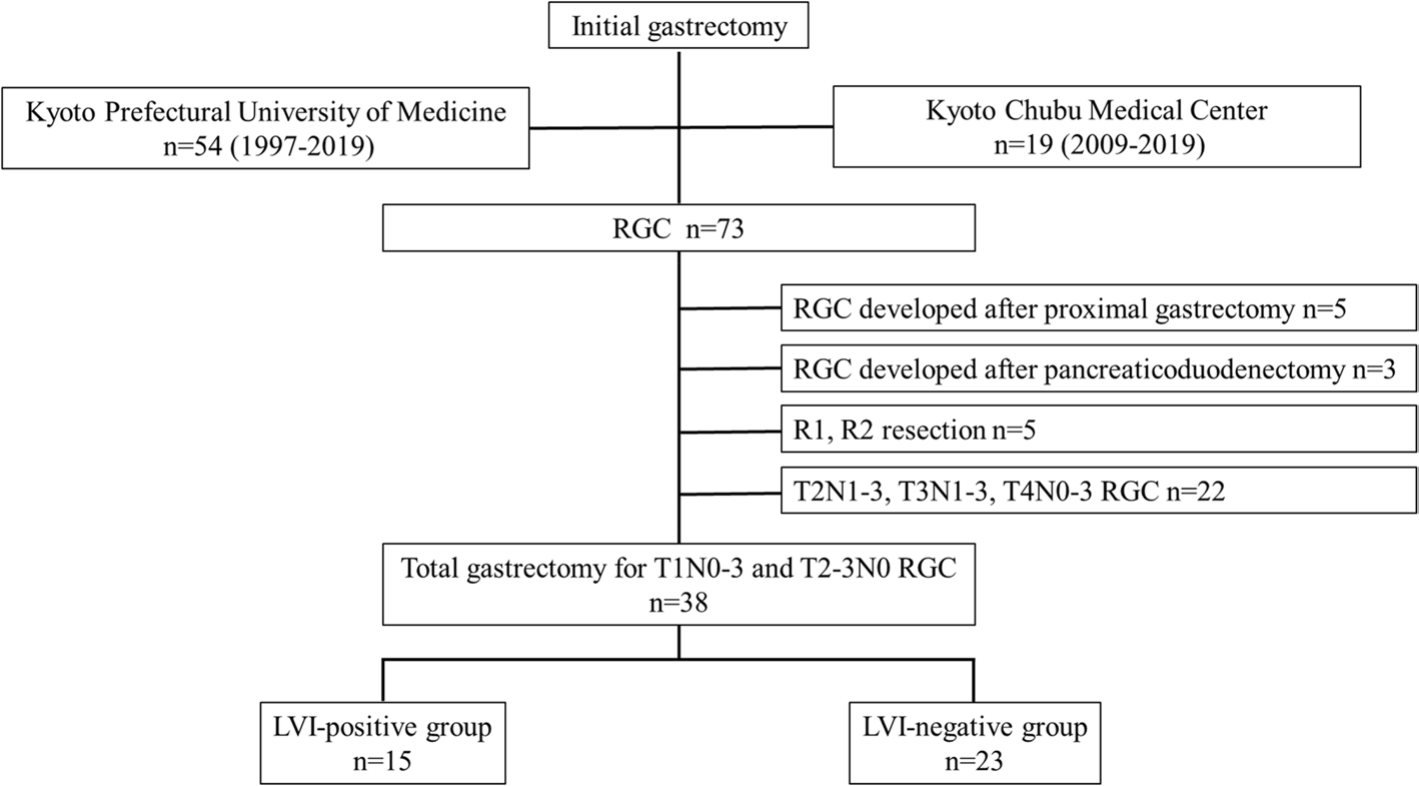
Flowchart for selecting and classifying subjects. Among the 73 consecutive patients with RGC who underwent curative surgery, 35 were excluded, and 38 were eligible for this study. Fifteen patients were classified into the LVI-positive group, and the remaining 23 were classified into the LVI-negative group. LVI, lymphovascular invasion; RGC, remnant gastric cancer

This study was conducted in accordance with the ethical principles of Kyoto Prefectural University of Medicine and the Declaration of Helsinki. Informed consent was obtained from all participants in the form of opt-outs on the hospital website. The experimental protocol was approved by the Ethical Review Board of Kyoto Prefectural University of Medicine (ERB-C-1414–1) and the Institutional Review Board of Kyoto Chubu Medical Center (C-326). We retrospectively analyzed the data of 73 consecutive patients who underwent curative resection for RGC at the Kyoto Prefectural University of Medicine between 1997 and 2019 and at the Kyoto Chubu Medical Center between 2009 and 2019. Since all included patients had primarily undergone pathologically curative surgery, no definite or conclusive recurrence was included in the analysis. Therefore, the diagnosis of RGC was made irrespective of the interval between the initial and second surgery. The patient underwent gastrectomy and lymph node dissection as per the guidelines of the Japanese Society of Gastric Cancer [13]. Patients with pT1N0-3 or pT2-3N0 RGC who underwent R0 resection after distal gastrectomy at the initial stage were selected. Ultimately, 38 patients with RGC were included in the study (Fig. 1).

Data on patient characteristics, pathological and surgical findings, and postoperative clinical course were obtained from the institution's medical records and databases. Physical examinations and blood tests, including those for tumor markers, were performed every three months, and computed tomography was performed every six months. Further treatment in cases of recurrence was decided based on the patient's consent, condition, and available evidence at that time.

### Histopathological evaluation

Surgical specimens underwent routine histopathological examinations and stained with hematoxylin and eosin. LVI was diagnosed based on the 8th edition of the TNM classification of malignant tumors [14]. For this study, pathology specimens predating the publication of the 8th edition of the TNM classification were reevaluated. Ly0 was defined as no lymphatic invasion, Ly1 as lymphatic invasion, V0 as no venous invasion, and V1 as microscopic venous invasion. In this study, LVI-negative (LVI [–]) was defined as Ly0, V0, or LVI-positive (LVI [ +]) as Ly1 and/or V1. Gastric cancer was classified according to the 15th edition of the Japanese Classification of Gastric Carcinomas [15]. Histological types were divided into two categories: differentiated (papillary, moderately, or well-differentiated adenocarcinoma) and undifferentiated (poorly or undifferentiated adenocarcinoma, signet-ring cell carcinoma, and mucinous adenocarcinoma).

### Statistical analyses

Data were analyzed using JMP version 10 (ASA Institute, Cary, NC, USA). To compare the clinicopathological characteristics between the two groups, chi-squared and Fisher’s exact probability tests were used for categorical variables, whereas Student’s t-tests and Mann–Whitney U tests were performed using unpaired continuous data. Survival curves were estimated using the Kaplan–Meier method, and differences were assessed using the log-rank test. Statistical significance was set at *P* < 0.05.

## Results

### Clinicopathological factors of patients with RGC

This study included 38 eligible patients with RGC who met the following criteria: no history of T2-3N1-3, T4N0-3, or R1-2 resection and no prior proximal gastrectomy or pancreaticoduodenectomy. Table 1 summarizes the patient characteristics, revealing a mean age of 73 years and a female-to-male ratio of 1:2.8. Among the 38 patients, 12 underwent surgery for benign diseases and 26 for gastric cancer, all of whom underwent an initial distal gastrectomy. The differences in initial disease did not affect clinicopathologic factors (Supplementary Table 1). All patients underwent total remnant gastrectomy with curative intent during laparotomy, and additional procedures, such as splenectomy, cholecystectomy, distal pancreatectomy, and partial colectomy, were performed based on intraoperative findings. Splenectomy was performed in eleven patients, cholecystectomy in eight, distal pancreatectomy in two, and partial colectomy in one. Reconstruction of the initial surgery was performed using Billroth I in 22 patients, Billroth II in 12, and Roux-en-Y in four. The RGCs were classified as pStage I in 28 patients and pStage II in 10 patients. Lymphatic and venous invasions were observed in 12 and 8 patients, respectively, with only lymphatic invasion in 7 cases, only venous invasion in 3 cases, and both lymphatic and venous invasions in 5 cases.

**Table 1:** Clinicopathological characteristics of patients with RGC

Variables		RGC	(*n* = 38)
Sex			
Female		10	(26%)
Male		28	(74%)
Age	Mean ± SD (years)	72.9 ± 9.1	
Body composition	Mean ± SD (kg/m^2^)	21.4 ± 3.2	
Initial gastric disease			
Benign		12	(32%)
Malignant		26	(68%)
Initial procedure			
Distal gastrectomy		38	(100%)
Interval between first and second surgeries			
Median (years)		19 (1–53)	
Reconstruction of initial surgery			
Billroth I		22	(58%)
Billroth II		12	(32%)
Roux-en-Y		4	(10%)
Depth of tumor			
T1		21	(55%)
T2		9	(24%)
T3		8	(21%)
Lymph node metastasis			
N0		36	(95%)
N1		0	(0%)
N2		2	(5%)
Stage			
I		28	(74%)
II		10	(26%)
Histological type			
Well		13	(35%)
Moderate		10	(26%)
Poor		8	(21%)
Sig		7	(18%)
Lymphatic invasion			
Negative		26	(68%)
Positive		12	(32%)
Venous invasion			
Negative		30	(79%)
Positive		8	(21%)
Surgical approach			
Laparotomy		38	(100%)
Surgical procedure			
Total gastrectomy		38	(100%)
Combined resection			
Spleen		11	(28%)
Gallbladder		8	(20%)
Distal pancreas		2	(5%)
Colon		1	(3%)
Operative time			
Median (min)		336 (174–612)	
Estimated blood loss			
Median (g)		550 (24–2512)	

*RGC* remnant gastric cancer, *SD* standard deviation

### Clinicopathological factors of LVI

Fifteen patients (39.5% [15/38]) were classified into the LVI-positive group, and the remaining 23 (60.5% [23/38]) were in the LVI-negative group. Univariate analysis revealed that LVI was significantly associated with lymph node metastasis (*P* < 0.049), lymphatic invasion (*P* < 0.001), and vascular invasion (*P* < 0.001). However, there were no correlations between the LVI status and variables such as sex, age, body composition, histological type, tumor size, tumor depth, staging, operative time, blood loss, intraoperative blood transfusion, or postoperative complications (Table 2). Postoperative complications included four cases of anastomotic leakage, two cases of pneumonia, one case of intra-abdominal abscess, and one case of pancreatic fistula. The most common form of recurrence in the LVI-positive group was lymph node metastasis (Supplementary Table 2).

**Table 2:** Clinicopathological factors of patients with or without LVI

	**T1N0-3, T2-3N0 RGC**				**Univariate**^**a**^
**Variables**	**LVI ( +)**	**(*n* = 15)**	**LVI (-)**	**(*n* = 23)**	***P*-value**
Sex					
Female	4	(40%)	6	(60%)	0.968
Male	11	(39%)	17	(61%)	
Age (years)					
< 65	2	(25%)	6	(75%)	0.335
> 65	13	(43%)	17	(57%)	
Body composition					
< BMI 25	13	(39%)	20	(61%)	0.979
> BMI 25	2	(40%)	3	(60%)	
Histological type					
Differentiated	10	(43%)	13	(57%)	0.530
Undifferentiated	5	(33%)	10	(67%)	
Tumor size					
< 50 mm	10	(34%)	19	(66%)	0.263
> 50 mm	5	(56%)	4	(44%)	
Depth of tumor					
T1	5	(24%)	16	(76%)	0.068
T2	6	(67%)	3	(33%)	
T3	4	(50%)	4	(50%)	
Lymph node metastasis					
N0	13	(36%)	23	(64%)	0.049^*^
N1	0	(0%)	0	(0%)	
N2	2	(100%)	0	(0%)	
Stage					
I	9	(32%)	19	(68%)	0.125
II	6	(60%)	4	(40%)	
Lymphatic invasion					
Negative	3	(12%)	23	(88%)	< 0.001^*^
Positive	12	(100%)	0	(0%)	
Venous invasion					
Negative	7	(23%)	23	(77%)	< 0.001^*^
Positive	8	(100%)	0	(0%)	
Operative time					
< 340 min	6	(32%)	13	(68%)	0.318
> 340 min	9	(47%)	10	(53%)	
Estimated blood loss					
< 400 mL	3	(21%)	11	(79%)	0.753
> 400 mL	12	(50%)	12	(50%)	
Intraoperative blood transfusion					
No	11	(37%)	19	(63%)	0.497
Yes	4	(50%)	4	(50%)	
Postoperative complication (CD grade ≥ 3)					
No	11	(37%)	19	(63%)	0.497
Yes	4	(50%)	4	(50%)	

*BMI* body mass index, *CD* Clavien–Dindo, *LVI* lymphovascular invasion, *RFS* recurrence-free survival, *RGC* remnant gastric cancer

^a^Chi-square test and Fisher’s exact test were used for univariate analysis

^*^P < 0.05: significantly different

### Analysis of prognostic factors

**Fig. 2:**
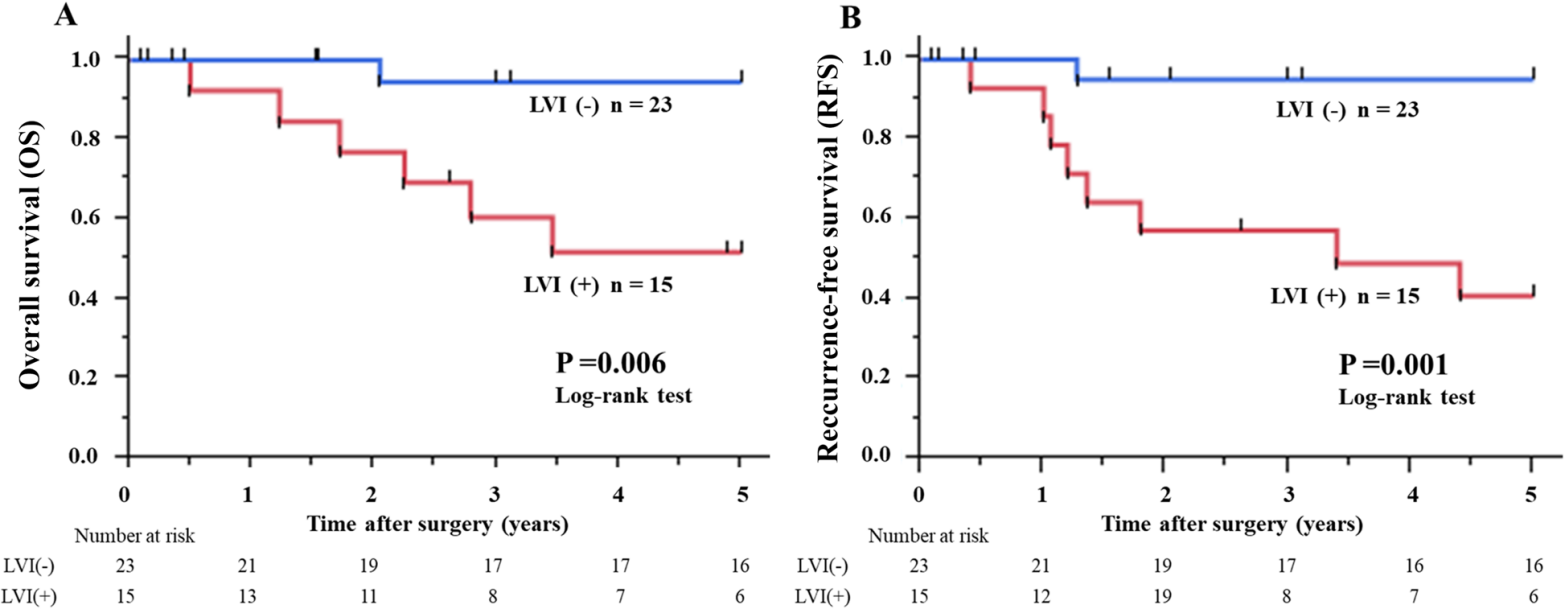
OS curves (**A**) and RFS curves (**B**) of T1N0-3 and T2-3N0 patients with RGC with or without pathological LVI. Patients with LVI had a significantly poorer prognosis in terms of the 5-year OS and RFS than those without LVI. OS, overall survival; LVI, lymphovascular invasion; RFS, recurrence-free survival; RGC, remnant gastric cancer

The median follow-up period was 5.34 years (interquartile range, 1.67–6.63). Figure 2 illustrates the overall survival (OS) and recurrence-free survival (RFS) curves of T1N0-3 and T2-3N0 RGC patients with and without LVI. The 5-year OS and RFS rates of the 23 patients with LVI-negative RGC were 94.4% and 95.0%, respectively. In contrast, the 5-year OS and RFS rates of the 15 patients with LVI-positive RGC were 51.9% and 44.0%, respectively. Patients with LVI had a significantly poorer prognosis for both OS (*P* = 0.006) and RFS (*P* = 0.001) than those without LVI. In the short-term results, there was no significant difference in 1-year OS (92.3% vs. 100.0%, *P* = 0.215), but a significant difference was observed in 3-year OS between patients with and without LVI (60.6% vs. 94.4%, *P* = 0.012). The Cox proportional hazards model showed that LVI was an independent factor affecting OS (*P* = 0.024; hazard ratio [HR], 8.27; 95% confidence interval [CI]: 1.285–161.6; Table 3) and RFS (*P* = 0.013; HR, 8.98; 95% CI: 1.513–171.2; Supplementary Table 3). Multivariate analysis was performed by excluding pN that correlated with pStage, as determined by Spearman's rank correlation coefficient, among the variables that were significantly different in the univariate analysis.

**Table 3:** Univariate and multivariate analyses of OS after surgery for RGC using Cox’s proportional hazard model

Variables	*n* = 38	Univariate^a^		Multivariate^b^		
		**5-year OS rate (%)**	***P*-value**	**HR**	**95%CI**	***P*-value**
Sex						
Female	10	77.8	0.944			
Male	28	77.0				
Age (years)						
< 65	8	85.7	0.512			
> 65	30	74.8				
Postoperative complication (CD grade ≥ 3)						
No	30	74.3	0.596			
Yes	8	85.7				
Tumor size						
< 50 mm	29	77.7	0.915			
> 50 mm	9	77.8				
Histological type						
Differentiated	23	67.6	0.130			
Undifferentiated	15	91.7				
pT						
pT1	21	83.3	0.387			
pT2-	17	69.1				
pN						
pN0	36	82.5	< 0.001*			
pN1-	2	0.0				
pStage						
pStage I	28	86.2	0.038*	1		
pStage II	10	51.4		2.37	0.498–12.74	0.273
LVI						
Negative	23	94.4	0.006*	1		
Positive	15	51.9		8.27	1.285–161.6	0.024*

*CD* Clavien–Dindo, *CI* confidence interval, *HR* hazard ratio, *LVI* lymphovascular invasion, *OS* overall survival

^a^Kaplan-Meier method; the log-rank test was used to detect statistical significance

^b^The Cox proportional hazards model was utilized in the multivariate analysis

^*^P < 0.05: significantly different

## Discussion

Our study revealed that LVI is a significant factor in determining OS and RFS in patients with RGC who are ineligible for adjuvant chemotherapy under the current treatment guidelines. Information on the long-term prognosis and associated clinicopathological factors of RGC is limited, and the treatment approach remains contentious [16, 17]. Previous studies have revealed postoperative complications [18], curative resections [3, 18], histopathological venous invasion [18, 19], TNM stage [19, 20], and lymph node ratio [21] as independent prognostic factors for OS. However, there is a lack of data on adjuvant chemotherapy for RGC, and its indications are unclear. This is the first study to highlight the importance of LVI in determining the long-term prognosis of patients with RGC who are not candidates for adjuvant chemotherapy. Therefore, this insight may help improve patient management and provide valuable insights for future research and clinical practice.

The presence of LVI has been shown to be a prognostic factor for many cancer types, including breast cancer [9], urothelial carcinoma [10], and colorectal cancer [12]. Although not recognized as a prognostic indicator in the TNM staging system of the Japanese and AJCC/UICC guidelines [13], several studies have shown that LVI may be an independent risk factor in patients with gastric cancer who are ineligible for adjuvant chemotherapy. Fujita et al. showed the clinical significance of vascular invasion as a prognostic factor in their study of pT1N + or pT2-3N0 gastric cancer [22]. Yagi et al. and Choi et al. showed that age and LVI are independent risk factors in early gastric cancer, respectively [23, 24]. Meanwhile, the predictive value of LVI as a prognostic factor in RGC remains unclear. LVI is considered an early stage of lymph node and distant metastasis, and its diagnosis suggests the presence of undetectable micrometastases, even in the absence of pathological lymph node metastasis or distant metastasis upon imaging [25]. In cases of RGC, the lymphatic flow differs from that in primary gastric cancer, owing to the initial surgery [26]. The extent of lymph node dissection is yet to be defined. LVI may suggest unexpected lymph node metastasis because RGC reportedly results in lymphatic flow to the mesentery of the jejunum and the mediastinal space beyond the normal extent of dissection [27, 28]. In our study, lymph node metastasis was the most common form of recurrence in LVI-positive cases. However, the number of cases of metastatic recurrence was small, and no significant differences in the form of recurrence were observed. Additionally, evaluating the survival time for each type of recurrence was also difficult.

The current Japanese Gastric Cancer Treatment Guidelines do not recommend adjuvant therapy for patients with pT1N0-3 or pT2–3N0 gastric cancer because of their excellent survival rates [13]. The indications for adjuvant chemotherapy in the current Japanese Gastric Cancer Treatment Guidelines are based on the results of the ACTS-GC trial [29], which included patients with stage II, IIIA, and IIIB cancers, excluding T1, as defined in the 13th edition of the Japanese classification [30]. This study population is consistent with "patients with stage IIA, IIB, IIIA, IIIB or IIIC (excluding T1 and T3)" as defined by the current Japanese classification [15] and TNM classification [14]. Therefore, the current guidelines recommend adjuvant chemotherapy for patients with stage II or III disease (except for pT1 or pT3N0). The indications for adjuvant chemotherapy for RGC are also unclear. However, in clinical practice, adjuvant chemotherapy for RGC is based on the current Japanese Gastric Cancer Treatment Guidelines. In literature, adjuvant chemotherapies such as S-1, uracil-tegafur, capecitabine plus oxaliplatin, and S-1 plus docetaxel have been administered to RGC patients with stage II or III cancers (excluding pT1 or pT3N0), with reference to clinical trials for gastric cancer [18, 21]. However, in this study, LVI was a more crucial prognostic factor than the pathological stage in the present subjects, and the results were different from the prognosis of gastric cancer of the same stage [22–24]. Lu et al. showed that the inclusion of LVI in the assessment of gastric cancer patients undergoing curative gastrectomy improved the accuracy of the TNM staging system [31]. Our findings support this concept and further suggest that LVI may be used to identify patients with pT1N0-3 or pT2–3N0 RGC who are at a high risk of recurrence. Therefore, evaluation of LVI may be useful in determining treatment strategies, including indications for adjuvant chemotherapy, for patients with pT1N0-3 or pT2-3N0 RGC.

The present study is subject to several limitations that warrant consideration. Firstly, its retrospective nature introduces inherent biases and limitations typical of retrospective studies. Additionally, the small sample size, particularly given the rarity of RGC at the specified stages, raises concerns regarding the risk of statistical overfitting. This risk is further compounded by the subset of patients with T1N0-3 or T2-3N0 RGC, representing a smaller subgroup within our cohort. The inclusion of older patients treated before 2000 may also introduce variability in treatment approaches and outcomes over time, further complicating the interpretation of our findings. Therefore, while our study provides valuable insights into the prognostic significance of LVI in this subgroup, caution should be exercised in interpreting the results due to the aforementioned limitations. Despite these limitations, our investigation identifies LVI as a potential prognostic factor in patients with T1N0-3 or T2-3N0 RGC, suggesting its potential usefulness in determining treatment strategies for patients with RGC. However, given the risk of statistical overfitting and the limitations inherent to our study design, larger prospective cohort studies are needed to validate these findings and establish their clinical application definitively.

## Conclusions

The investigation found that LVI was a prognostic factor in patients with T1N0-3 or T2-3N0 RGC. Evaluation of LVI may be useful in determining treatment strategies for patients with RGC.

### Supplementary Information

**Additional file 1:** **Supplementary Table 1.** The clinicopathological factors according to the benign or malignant of the first surgery in patients with RGC. **Supplementary Table 2.** Comparison of recurrence rates and patterns according to LVI status. **Supplementary Table 3.** Univariate and multivariate analyses of RFS after surgery for RGC using Cox’s proportional hazard model.

## Data Availability

No datasets were generated or analysed during the current study.

## Abbreviations

RGC: Remnant gastric cancer
LVI: Lymphovascular invasion
OS: Overall survival
RFS: Recurrence-free survival
SD: Standard deviation
BMI: Body mass index
CD: Clavien–Dindo
CI: Confidence interval.
HR: Hazard ratio
