# Supplementary material for: Lymphovascular invasion is associated with poor long-term outcomes in patients with pT1N0-3 or pT2-3N0 remnant gastric cancer: a retrospective cohort study
Source: World J Surg Oncol. 2024 Apr 5;22:86. doi: 10.1186/s12957-024-03371-z (PMC10996196; doi:10.1186/s12957-024-03371-z)
Supplement: Supplementary file 1 — Additional file 1: Supplementary Table 1. The clinicopathological factors according to the benign or malignant of the first surgery in patients with RGC. Supplementary Table 2. Comparison of recurrence rates and patterns according to LVI status. Supplementary Table 3. Univariate and multivariate analyses of RFS after surgery for RGC using Cox’s proportional hazard model. [file 12957_2024_3371_MOESM1_ESM.zip › Supplementary/Supplementary_Table_2_revised.docx]

**Supplementary Table 2. Comparison of recurrence rates and patterns according to LVI status**

|  | **Variables** | | **T1N0-3, T2-3N0 RGC** | | | | **P-value** |  |
| --- | --- | --- | --- | --- | --- | --- | --- | --- |
|  |  |  | **LVI (+)** | **(n=15)** | **LVI (-)** | **(n=23)** |  |  |
|  | Recurrence | | 7 | (46.7%) | 1 | (4.3%) | 0.001* |  |
|  | Recurrence pattern | |  |  |  |  | 0.530 |  |
|  |  | Local | 0 | (0.0%) | 0 | (0.0%) |  |  |
|  |  | Peritoneal | 1 | (14.3%) | 0 | (0.0%) |  |  |
|  |  | Hematogenous | 2 | (28.6%) | 1 | (100.0%) |  |  |
|  |  | Lymph node | 3 | (42.9%) | 0 | (0.0%) |  |  |
|  |  | Mixed | 1 | (14.3%) | 0 | (0.0%) |  |  |

LVI, lymphovascular invasion; RGC, remnant gastric cancer.

* P < 0.05, significant difference.
